# Genome-Wide Identification and Expression Analysis of the YTH Domain-Containing Protein Gene Family in *Salvia miltiorrhiza*

**DOI:** 10.3390/ijms26104645

**Published:** 2025-05-13

**Authors:** Chunling Wang, Yunliang Peng, Xian Pan, Sixuan Zhang, Yayun Xu, Caili Li, Butuo Zhu, Lili Niu, Shanfa Lu

**Affiliations:** 1 State Key Laboratory for Quality Ensurance and Sustainable Use of Dao-di Herbs, Institute of Medicinal Plant Development, Chinese Academy of Medical Sciences & Peking Union Medical College, Beijing 100193, China; clingwang@implad.ac.cn (C.W.); pengyunliang@implad.ac.cn (Y.P.);; 2 Key Laboratory of Chinese Medicine Resources Conservation, State Administration of Traditional Chinese Medicine of the People’s Republic of China, Institute of Medicinal Plant Development, Chinese Academy of Medical Sciences & Peking Union Medical College, Beijing 100193, China

**Keywords:** *Salvia miltiorrhiza*, YTH domain, m^6^A, gene family, plant growth regulator

## Abstract

YTH domain-containing proteins act as the primary readers of *N*^6^-methyladenosine (m^6^A), playing an important role in plant development and stress responses. However, little is known about the YTH proteins in medicinal plants. Genome-wide identification of the *YTH* gene family in the medicinal model plant, *Salvia miltiorrhiza* Bunge, identified a total of nineteen *SmYTH* genes from five chromosomes, with *SmYTH8*–*SmYTH19* clustered on chromosome 8. Phylogenetic analysis showed that SmYTH proteins belong to the YTHDF category. No YTHDC members were identified. Conserved domain identification, amino acid sequence alignment, and phase separation prediction revealed that SmYTH1–SmYTH4 exhibited the characteristic m^6^A reader protein feature, containing conserved aromatic cages (WWW) capable of binding m^6^A residues. SmYTH5–SmYTH19 proteins contain a unique conserved F-box protein interaction domain that has not been reported previously. qRT-PCR analysis revealed tissue-specific patterns, with *SmYTH1*–*SmYTH4* genes highly expressed in roots and leaves, whereas *SmYTH8*–*SmYTH19* were mainly expressed in leaves. The results were consistent with RNA-seq data. The expression of various *SmYTHs* and the content of phenolic acid active ingredients were significantly altered under MeJA and SA treatments. The results provide useful information for further studies on the biological functions of m^6^A and YTH proteins in *S. miltiorrhiza*.

## 1. Introduction

Over 160 RNA chemical modifications have been identified, with *N^6^*-methyladenosine (m^6^A) being the most extensively studied in plants and animals [1]. Plant m^6^A methylation can be dynamically added, removed, and specifically recognized by methyltransferases (“writer”), demethylases (“eraser”), and m^6^A-binding proteins (“reader”) [2,3,4,5,6,7,8,9,10,11]. Among them, m^6^A-binding proteins precisely regulate RNA processing and metabolism, including RNA stability, translation efficiency, alternative splicing, polyadenylation, nuclear export, and so on [12,13,14,15,16,17,18,19,20], and are involved in plant morphogenesis, flowering, ABA response, pathogen defense, and stress responses [2,4,21,22,23,24,25,26,27,28,29,30]. Identification and characterization of m^6^A reader proteins is essential for elucidating the underlying mechanisms of m^6^A-mediated regulation in vivo.

The first identified animal m^6^A reader proteins were five YT521-B homology (YTH) domain-containing proteins, including YTHDF1, YTHDF2, YTHDF3, YTHDC1, and YTHDC2 [31,32,33,34]. They feature a conserved aromatic cage (WWW) for m^6^A-binding and/or prion-like domains (PrLD) that promote liquid–liquid phase separation [17,19,35]. So far, all of the m^6^A reader proteins identified in plants belong to the YTH family. There are thirteen *YTH* genes (*AtECT1-12* and *AtCPSF30*) in *Arabidopsis* [36]. Among them, *AtECT2/3/4* and *AtCPSF30* regulate trichome branching, flowering time, leaf growth, cell proliferation, nitrate transport, and abscisic acid (ABA) response [15,21,25,26,28,29,37]. *AtECT1*, *AtECT8*, and *AtECT12* are involved in stress responses through phase separation [38,39,40,41]. In addition to *Arabidopsis*, YTH proteins also play critical roles in other plants. For instance, apple MhYTP2 enhances resistance to powdery mildew [30]. Rice YTH07 promotes flowering by reducing protein levels of the flowering repressor OsCOL4 through phase-separated condensates [42]. *Foxtail millet* SiYTH1 stabilizes ROS-related transcripts to enhance drought resistance under stress conditions [43]. Tomato SlYTH2 regulates the translation efficiency of volatile aroma-related target genes through forming RNA protein condensates, which subsequently influence flavor [44]. Despite these significant advancements, the *YTH* gene family in many other plants, such as medicinal plant species, remains poorly understood.

*Salvia miltiorrhiza* Bunge, a well-known traditional Chinese medicinal plant and a model medicinal plant, is abundant in bioactive compounds, including tanshinones and salvianolic acids [45]. It is widely used to treat cardiovascular diseases and various other diseases. Identification and characterization of genes associated with *S. miltiorrhiza* development, stress responses, and bioactive compound biosynthesis have attracted widespread interest [45]. The involvement of transcription factors, microRNAs, and kinases in the regulation of bioactive compound biosynthesis in *S. miltiorrhiza* have been intensely studied [46,47,48,49,50,51,52]. However, there is no information on the regulation of bioactive compound biosynthesis at the RNA level. m^6^A is one of the main RNA epigenetic modifications in plants and a hot research area of epigenetics. It is a novel regulatory mechanism involved in plant development, stress responses, and probably secondary metabolism. As the first step to elucidate the regulatory role of m^6^A in *S. miltiorrhiza*, genome-wide identification of *S. miltiorrhiza YTH* genes (*SmYTHs*) was carried out. The characteristics of these genes and their deduced proteins were subsequently analyzed in detail. Conserved and unique features were revealed. In addition, gene expression patterns and phenolic acid contents in *S. miltiorrhiza* roots and leaves treated with MeJA and SA were determined. The results provide useful information for further studies on the biological functions of m^6^A and its reader proteins in *S. miltiorrhiza*, particularly in the biosynthesis of bioactive compounds.

## 2. Results

### 2.1. The YTH Gene Family in S. miltiorrhiza

Blast analysis of *Arabidopsis* and *Oryza sativa* YTH protein sequences against the *S. miltiorrhiza* line shh genome with an *e*-value cutoff of 1 × 10^−5^ identified 19 putative *SmYTH* genes, designated as *SmYTH1* to *SmYTH19*. The gene ID, chromosome start and end positions, amino acid count, molecular weight (Mw), and isoelectric point (p*I*) of the identified *SmYTHs* are shown in Table 1. The sequences of all SmYTH proteins are provided in Supplementary Table S1. Chromosome localization analysis showed that *SmYTH* genes were unevenly distributed across the chromosomes. There was one on each of chromosome 1, chromosome 5, and chromosome 7, two on chromosome 3, and 10 on chromosome 8.

### 2.2. Evolutionary Relationship of SmYTH Proteins

**Figure 1:**
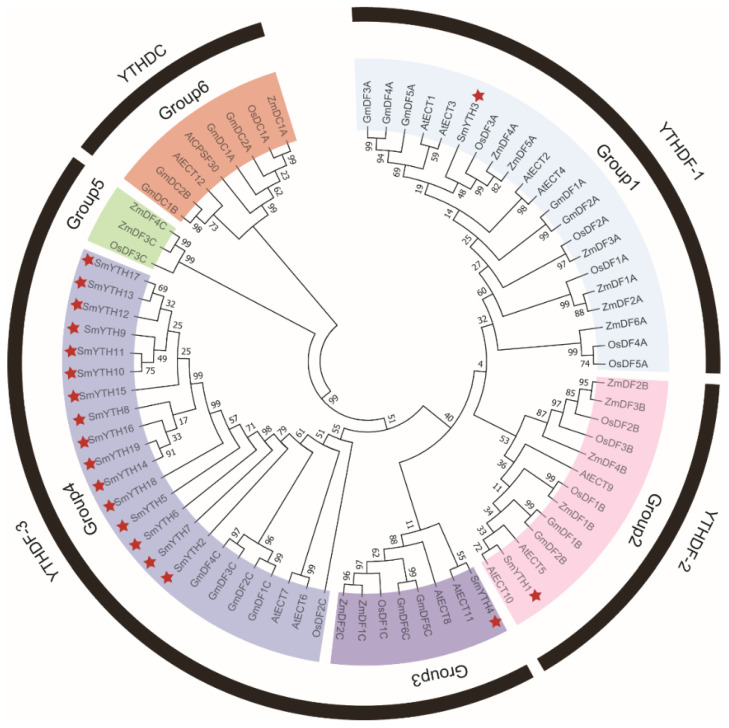
Phylogenetic tree of SmYTH proteins from *Arabidopsis*, soybean, rice, and maize. The evolutionary relationships show that the YTH proteins from these plant species could be divided into four clades and six groups. Among them, Group 1 belongs to YTHDF-1, Group 2 belongs to YTHDF-2, Groups 3, 4, and 5 belong to YTHDF-3, and Group 6 belongs to YTHDC. SmYTH proteins are labeled with a red pentagram.

To analyze the evolutionary relationship of SmYTH proteins, a phylogenetic tree was constructed for 76 YTH proteins from *Arabidopsis*, soybean, and rice using the neighbor-joining (NJ) method in MEGA11 [53]. As shown in Figure 1, YTH proteins could be classified into four clades, including YTHDF-1, YTHDF-2, YTHDF-3, and YTHDC. All of the SmYTHs belonged to the YTHDF clades, with the majority being members of the YTHDF-3 clade. No YTHDC clade members were identified in *S. miltiorrhiza*, which is different from the other four plant species that had members in four clades.

### 2.3. Gene Structure, Conserved Domain, and Conserved Motif of SmYTHs

**Figure 2:**
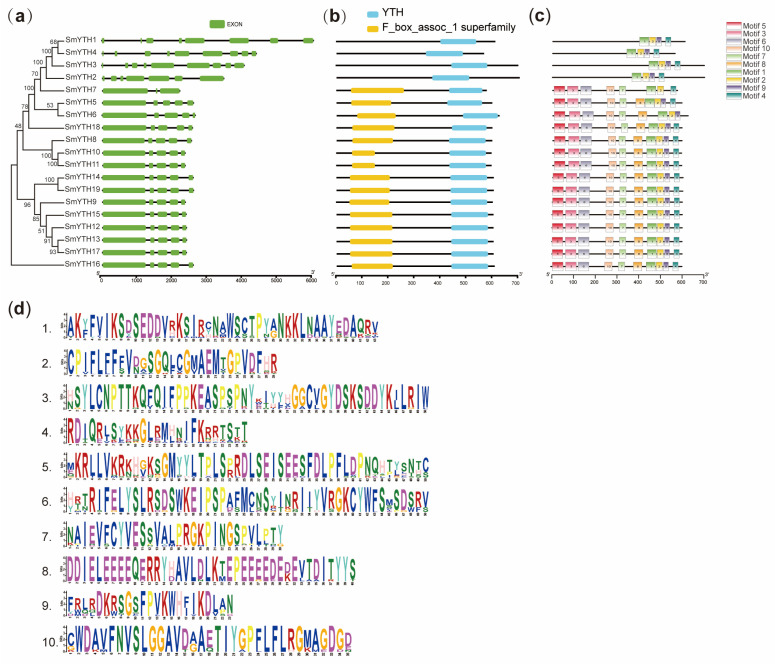
Gene structures, conserved YTH domains, and motifs of SmYTH proteins. (**a**) Exon/intron organization of *SmYTH* genes. Green boxes represent exons, and black lines represent introns. (**b**) The YTH domain of SmYTH proteins. Blue boxes represent YTH domains. Yellow boxes represent F-box interaction domains. (**c**) Distribution of conserved motifs in SmYTH proteins. Ten putative motifs are indicated in different colored boxes. (**d**) Detailed sequence logo analysis of the conserved motifs in SmYTH proteins. Different letters and font colors represent different aminos, with base size reflecting the frequency of occurrence.

Gene structure analysis revealed that all *SmYTH* family members were split genes, each of which contained at least two introns (Figure 2a). *SmYTH1*, *SmYTH2*, *SmYTH3*, and *SmYTH4* exhibited the most complex structures with respect to gene size and the arrangement of exons and introns. *SmYTH7* had the simplest structure with only two introns. *SmYTH5*, *SmYTH6*, and *SmYTH8*–*SmYTH19* shared similar gene structures (Figure 2a). A search using the Batch CD Search tool in the Conserved Domain Database (CCD) showed the existence of a typical functional YTH domain in each SmYTH protein (Figure 2b). This domain was located near the C-terminal. Except for SmYTH1–SmYTH4, all other SmYTHs contained a conserved F-box protein interaction domain (F_box_assoc_1), which has not been found in other reported plant YTHs. This box could be associated with some specific functions of SmYTHs, which need to be further investigated. A total of ten conserved motifs were identified in SmYTH proteins using MEME (Figure 2c). The length of these motifs varied from 23 to 50 aa. Details of the motifs are displayed in Figure 2d.

### 2.4. Chromosome Location of SmYTH Genes and Collinear Relationships

**Figure 3:**
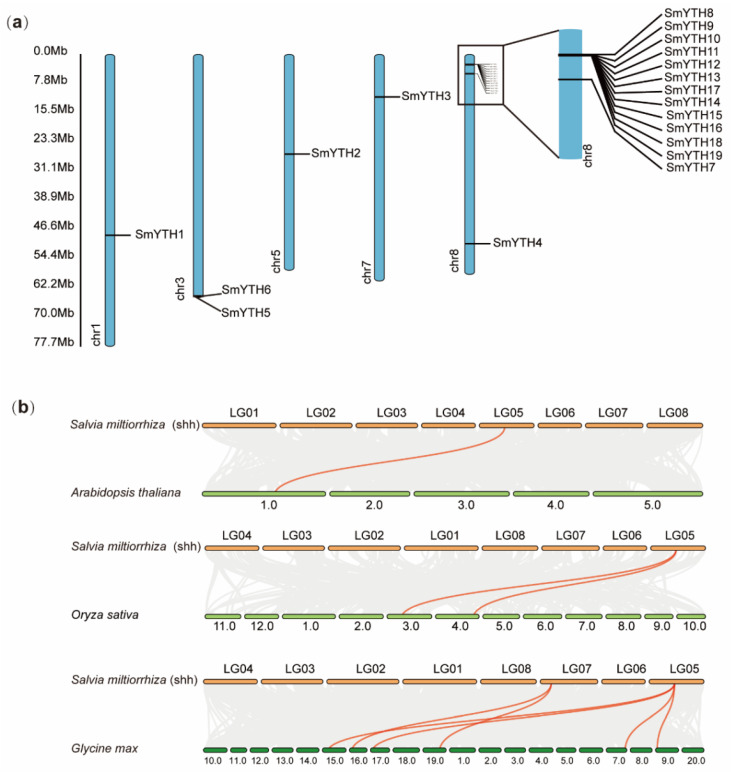
Chromosome location and synteny relationship analysis of *SmYTH* genes. (**a**) Location of *SmYTH* genes in the assembled pseudo-chromosomes of *S. miltiorrhiza* line shh. Chromosome number is indicated at the bottom of each chromosome. *SmYTH7*–*SmYTH19* on chromosome 8 are also shown in an enlarged box. (**b**) Synteny gene analysis of *YTHs* from *S. miltiorrhiza* and other plant species, including *Arabidopsis*, *O. sativa*, *G. max*, and *Zea mays*. Gray lines in the background indicate the collinear blocks within the genomes of *S. miltiorrhiza* and other plants. Red lines indicate the synteny gene pairs of *YTH* genes.

The 19 *SmYTH* genes were distributed across 5 chromosomes of *S. miltiorrhiza* (Figure 3a). Specifically, *SmYTH1* was located on chromosome 1 (chr01), *SmYTH2* on chromosome 5 (chr05), *SmYTH3* on chromosome 7 (chr07), and *SmYTH4* and *SmYTH7* on chromosome 8 (chr08). *SmYTH5* and *SmYTH6* were adjacent to each other on chromosome 3 (chr03). The other 12 genes, including *SmYTH8*–*SmYTH19*, were clustered on chromosome 8 (Figure 3a). In order to further explore the phylogenetic relationship of SmYTHs and YTHs from other plant species, comparative collinear maps were constructed. The results showed that *SmYTH2* exhibited synteny with *Arabidopsis AT1G48110*, *Oryza sativa Os03t0317000* and *Os04t0608800*, and four *Glycine max GmYTHs*, including *KRH11876*, *KRH02429*, *KRH50636*, and *KRH36908*. *SmYTH3* shared collinearity with *G. max KRH06717* and *KRG94807* (Figure 3b). It suggests that *SmYTH2* and *SmYTH3* likely existed prior to ancestral divergence of these plants, whereas other *SmYTH* genes could have arisen through gene duplication or segmental duplication events after ancestral divergence.

### 2.5. Cis-Acting Elements in the Promoter Region of SmYTH Genes

**Figure 4:**
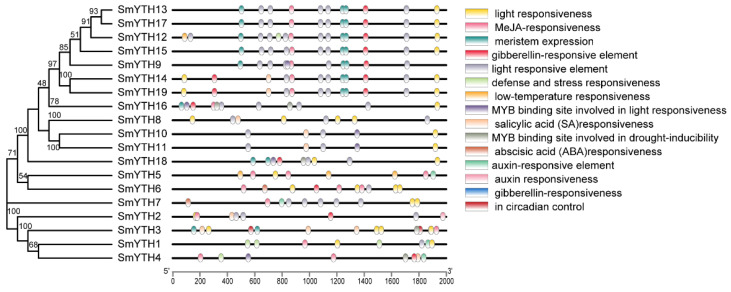
Promoter *cis*-elements analysis of *SmYTH* genes. The 2 kb DNA fragment upstream of the transcription start site of each *SmYTH* gene is shown.

*Cis*-acting regulatory element analysis showed that there were multiple hormone- and plant-growth-regulator (PGR)-related elements existing in the promoter of *SmYTH* genes (Figure 4). They included methyl-jasmonate (MeJA)-responsive elements, salicylic-acid (SA)-responsive elements, abscisic-acid (ABA)-responsive elements, and gibberellin (GA)-responsive elements (Figure 4). The existence of these elements indicates the importance of *SmYTH* genes in hormone signaling pathways.

### 2.6. The Structure of SmYTH Proteins

**Figure 5:**
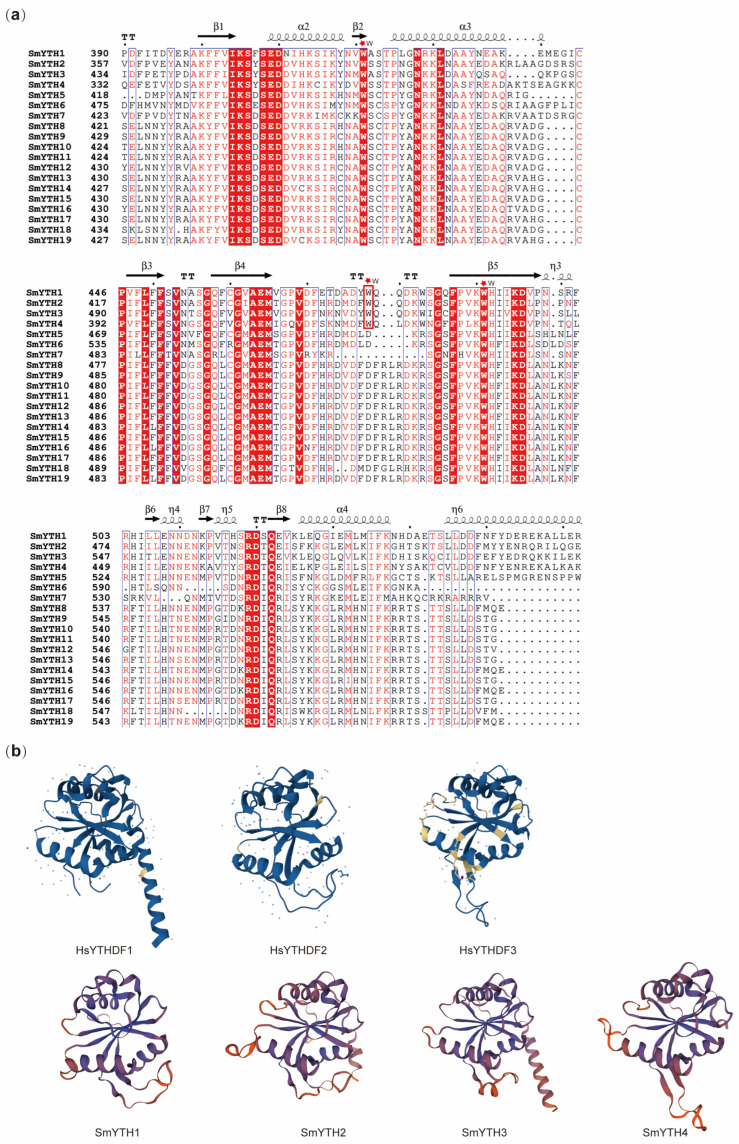
Secondary structure and three-dimensional structure prediction of SmYTH proteins. (**a**) Sequence alignment of the SmYTH family proteins. The conserved amino acids are colored as following: similar residues are depicted as red letters and identical residues as red boxes. The positions marked by red pentagrams are three conserved tryptophan residues (WWW) of SmYTH1–SmYTH4 proteins. (**b**) Predicted three-dimensional structures of SmYTH1–SmYTH4 based on homology modeling of human HsYTHDF1–HsYTHDF3. The blue dots represent some unknown atoms or ions.

The amino acid composition and arrangement order of protein molecules cannot fully explain their biological activity and physicochemical properties determined by their higher-order structures. Analysis of the secondary structure of SmYTH proteins showed that the YTH domain contained highly conserved amino acid residues, and almost every SmYTH protein had four to five α-helices and eight β-folds (Figure 5a).

It has been reported that the YTH domain of YTHDFs features a WWW cage and the YTH domain of YTHDCs has a WWL/W/Y cage. These cages recognize the methyl moiety of m^6^A [31,54]. Three-dimensional structure analysis of SmYTHs showed that the aromatic cages composed of WWW existed in SmYTH1–SmYTH4. The other SmYTH proteins only had WW amino acid residues (Figure 5a). Construction of the spatial structure of SmYTH1–SmYTH4 proteins based on homology modeling showed that SmYTH1–SmYTH4 and YTHDF proteins shared similar three-dimensional structures, exhibiting a globular fold with a four-stranded sheet center encircled by four helices, flanking regions on both sides, and a central core (Figure 5b). Each SmYTH protein contained multiple α-helices and β-folds on the outside or inside of the three-dimensional (3D) structure (Figure 5b). This is consistent with the secondary structure of other conserved YTHs (Figure 5a). In mammals, the center of YTHs surrounded by these secondary structures forms the binding site of m^6^A [32]. The SmYTH models also exhibited the binding sites in the center of these folds, which were similar to the spatial structure of mammalian YTH proteins (Figure 5b). The results indicate that SmYTH1–SmYTH4 proteins contained the m^6^A-binding sites and could function through binding m^6^A.

### 2.7. SmYTH1–SmYTH4 in Phase Separation

**Figure 6:**
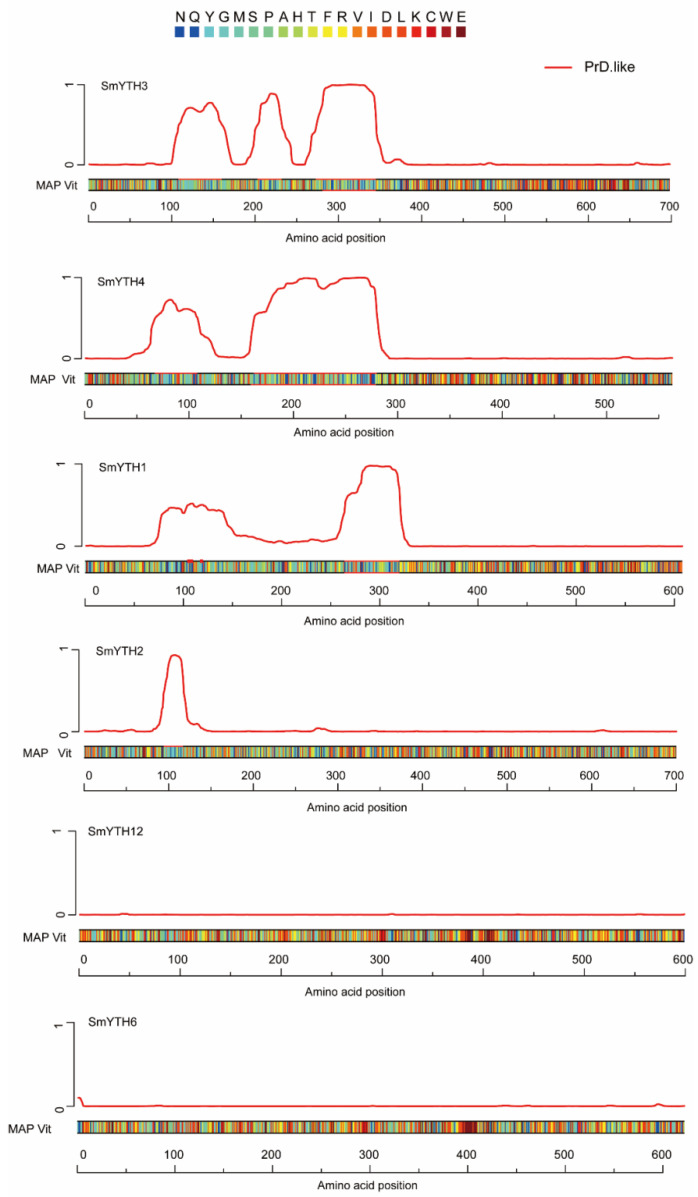
PrLD and disordered region predictions of SmYTH proteins in the “prion-like amino acid composition” (PLAAC; http://plaac.wi.mit.edu/, accessed on 27 September 2024). SmYTH6 and SmYTH12 are representatives of SmYTH5–SmYTH19.

Considering that human m^6^A reader proteins YTHDF1–YTHDF3 can undergo LLPS, a process enhanced by multivalent m^6^A modifications [17,19,35], we investigated whether SmYTH proteins contained the prion-like domains (PrLDs), which are known to drive proteins to undergo phase separation [55]. The results showed that SmYTH1–SmYTH4 proteins contained the disordered PrLDs at their N-termini, whereas the other SmYTH proteins lacked such a domain (Figure 6). This suggests that SmYTH1–SmYTH4, particularly SmYTH3 that has three PrLDs, may undergo phase separation in a manner similar to human YTHDF1–YTHDF3 proteins.

### 2.8. Differential Expression of SmYTH Genes in S. miltiorrhiza

**Figure 7:**
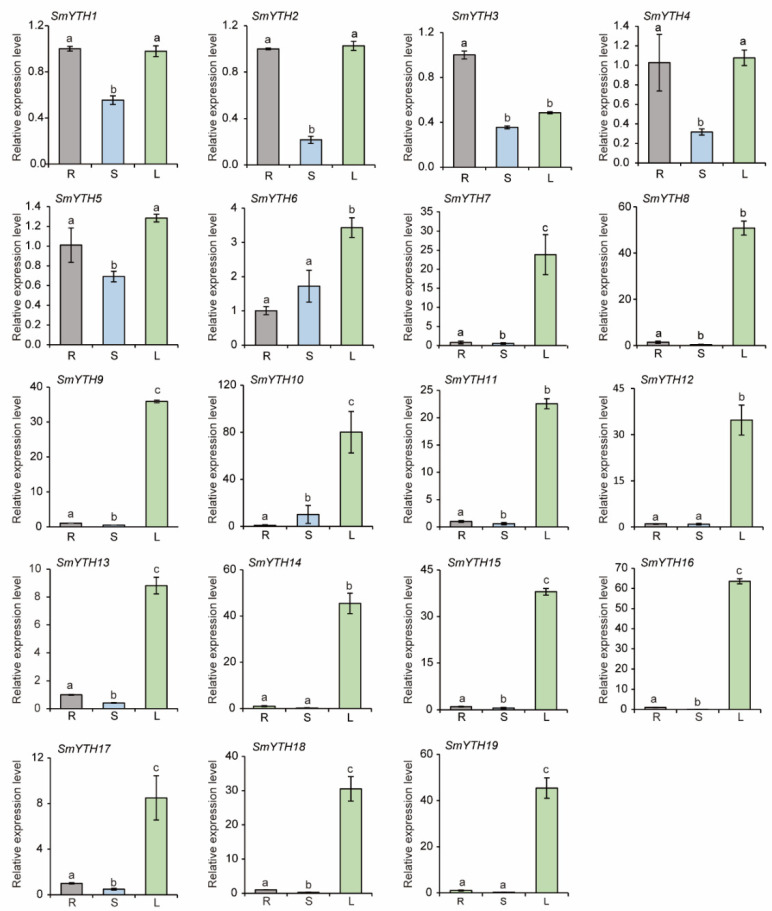
Expression patterns of *SmYTH* genes in roots (R), stems (S), and leaves (L) of *S. miltiorrhiza*. *SmUBQ* was used as the normalization control. The values represent the mean ± SD of three biological and three technical replicates. Different lowercase letters above the bars indicate significant differences (*p* < 0.05) based on one-way ANOVA.

Given that tissue-specific gene expression often reflects their biological functions, we analyzed the expression patterns of *SmYTH* genes in roots, stems, and leaves of *S. miltiorrhiza* plants using the qRT-PCR method. The results showed that *SmYTH1*–*SmYTH6* genes were ubiquitously expressed in roots, stems, and leaves (Figure 7). Among them, *SmYTH3*, a homolog of *AtECT2* in *Arabidopsis*, was expressed relatively high in roots. *SmYTH1*, *SmYTH2*, *SmYTH4*, and *SmYTH5* exhibited relative high expression in roots and leaves. *SmYTH6* showed relative high expression in leaves (Figure 7). Differently, *SmYTH7* and *SmYTH8*–*SmYTH19* on chromosome 8 were predominantly expressed in leaves (Figure 7). The results indicate that *SmYTH1*–*SmYTH6* probably play diverse biological roles in different organs, while *SmYTH7* and *SmYTH8*–*SmYTH19* mainly function in leaf-related processes.

**Figure 8:**
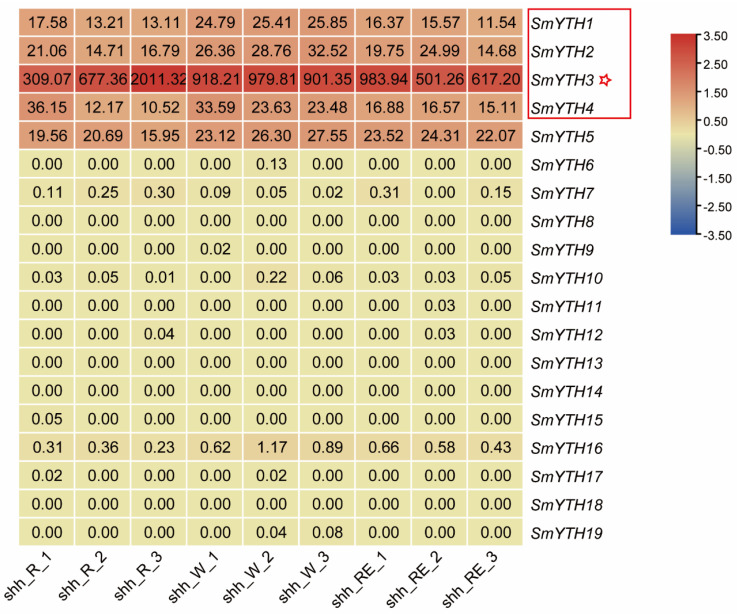
Transcriptomic analyses of *SmYTH* genes in the roots of *Salvia miltiorrhiza* line shh. R: red root, W: white root, and RE: root epidermis. The asterisk represents *SmYTH3*, which had the highest expression level in roots. *SmYTH1*, *SmYTH2*, and *SmYTH4* in the box were also expressed in the roots analyzed.

Since the root is the medicinal part of *S. miltiorrhiza* and one of the major classes of medicinal ingredients, tanshinones, are accumulated in the epidermis of red roots, we further analyzed the expression of *SmYTHs* in young white roots, mature red roots, and the epidermis of red roots using high-throughput RNA-seq data from *S. miltiorrhiza* [56]. The results showed that *SmYTH1*–*SmYTH5* were expressed in the root tissues analyzed, whereas the expression of *SmYTH6–SmYTH19* in these tissues was extremely low (Figure 8). The results were consistent with qRT-PCR analysis (Figure 7). In addition, among the five *SmYTH* genes expressed in roots, *SmYTH3* exhibited the highest expression (Figure 8). These results indicate the importance of the five genes, particularly *SmYTH3*, in root growth, development, or physiological and biochemical processes.

**Figure 9:**
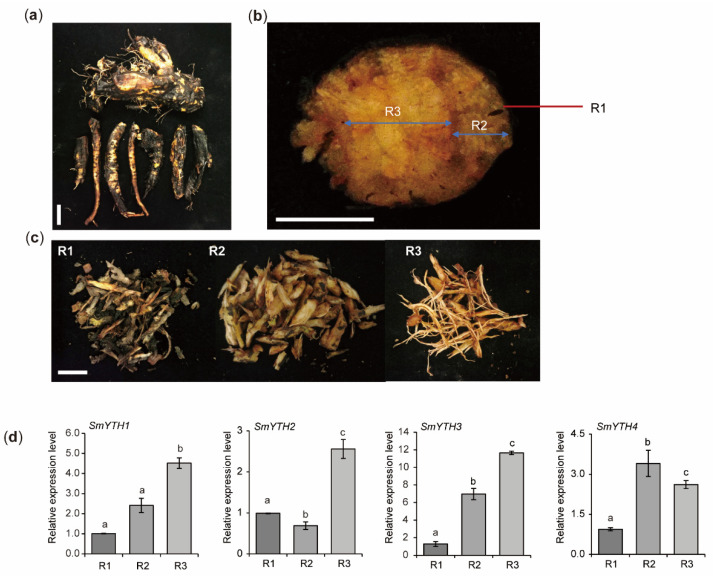
Expression analysis of *SmYTH1–SmYTH4* in the epidermis, phloem, and xylem of mature root tissues. (**a**) Morphology of mature roots of three-year-old *S. miltiorrhiza* line shh. Bar = 1 cm. (**b**) Cross-section of the mature roots. (**c**) The isolated epidermis, phloem, and xylem of roots. R1: epidermis, R2: phloem, and R3: xylem. Bar = 1 cm. (**d**) Real-time qRT-PCR analysis of *SmYTH1–SmYTH4* gene expression in the epidermis, phloem, and xylem, respectively. *SmUBQ* was used as an internal reference control. Each value represents the mean ± SD of three biological and technical replicates. Different lowercase letters above the bars indicate significant differences (*p* < 0.05) based on one-way ANOVA.

To further explore the function of *SmYTH1*–*SmYTH4* in roots, we isolated the epidermis, phloem, and xylem of mature roots of three-year-old *S. miltiorrhiza* plants and analyzed gene expression in these tissues. The results showed that all of them were expressed in the three tissues analyzed, with relatively lower expression levels in root epidermis. *SmYTH1*–*SmYTH3* exhibited the highest expression in root xylem, whereas *SmYTH4* showed the highest expression in root phloem (Figure 9). Differential expression of these genes could be associated with their function in the formation of different root tissues or the biosynthesis of different metabolites.

### 2.9. Subcellular Localization of SmYTH3

**Figure 10:**
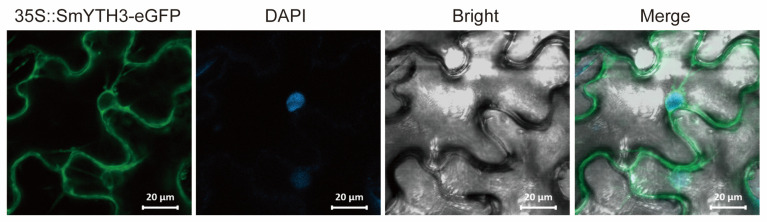
Subcellular localization analysis of SmYTH3. The 1305::*SmYTH3-eGFP* was transiently expressed through agroinfiltration in *Nicotiana benthamiana* leaves. Green fluorescence of the eGFP was viewed using confocal laser microscopy. DAPI, 4′,6-diamidino-2-phenylindole, a fluorescent dye binding to DNA; eGFP, GFP fluorescence; Bright, bright-field image. Scale bar = 10 μm.

The subcellular localization of a protein is closely related to its function. In order to know the site where SmYTHs exert their activity, *SmYTH3*, which is highly expressed in roots, was selected for subcellular localization analysis. *SmYTH3-eGFP* was transiently expressed in tobacco leaf cells. Analysis of the eGFP green fluorescence using confocal laser microscopy showed that the eGFP fluorescence signal was primarily detected in the cytoplasm and on membranes (Figure 10). No signals were found in the nucleus (Figure 10).

### 2.10. PGR Responses of SmYTHs in S. miltiorrhiza Plantlets and Hairy Roots

Previous studies showed that *S. miltiorrhiza* was highly sensitive to the treatment of hormones and PGRs, such as MeJA and SA, which play crucial regulatory roles in bioactive compound biosynthesis [45,57,58]. Given that the promoter of *SmYTHs* contains hormone/PGR-responsive elements (Figure 4), we investigated the expression patterns of representative *SmYTH* genes, including *SmYTH1*–*SmYTH6*, under MeJA and SA treatments.

**Figure 11:**
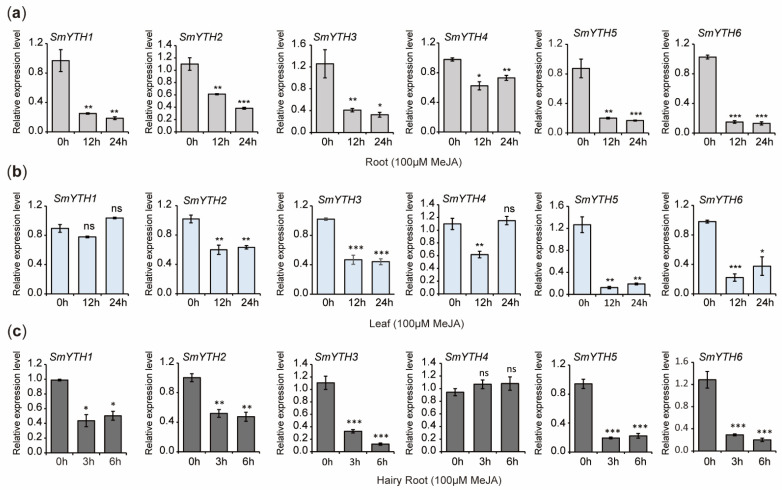
Expression of *SmYTH1*–*SmYTH6* in roots and leaves of *S. miltiorrhiza* plantlets and two-month-old hairy roots treated with MeJA. (**a**) Expression of *SmYTH1*–*SmYTH6* in roots treated with 100 μM of MeJA for 0 h, 12 h, and 24 h. (**b**) Expression of *SmYTH1*–*SmYTH6* in leaves treated with 100 μM of MeJA for 0 h, 12 h, and 24 h. (**c**) Expression of *SmYTH1*–*SmYTH6* in hairy roots treated with 100 μM of MeJA for 0 h, 3 h, and 6 h. *SmUBQ* was used as the normalization control. Each value represents the mean ± SD of three biological and three technical replicates. The asterisks represent significant differences between the test group and the control group (0 h) (* *p* < 0.05, ** *p* < 0.01, and *** *p* < 0.001, Student’s *t* test), and ‘ns’ indicates no significant difference.

The results indicated that the expression of *SmYTH1*–*SmYTH6* in roots and the expression of *SmYTH2*, *SmYTH3*, *SmYTH5*, and *SmYTH6* in leaves was significantly downregulated after 12 and 24 h of MeJA treatment. The expression of *SmYTH1* in leaves remained unaffected. *SmYTH4* was downregulated at 12 h but returned to the baseline level at 24 h (Figure 11a,b). In hairy roots, MeJA treatment significantly suppressed the expression of *SmYTH1*–*SmYTH3*, *SmYTH5*, and *SmYTH6* (Figure 11c). The results suggest that the majority of *SmYTH* genes in both plantlets and hairy roots were highly responsive to MeJA treatment.

**Figure 12:**
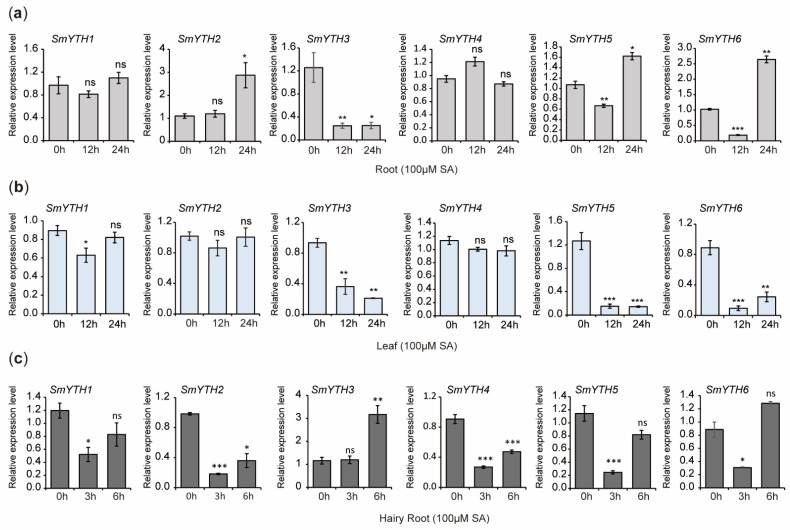
Expression of *SmYTH1*–*SmYTH6* in roots and leaves of *S. miltiorrhiza* plantlets and two-month-old hairy roots treated with SA. (**a**) Expression analysis of *SmYTH1*–*SmYTH6* in roots treated with 100 μM of SA for 0 h, 12 h, and 24 h. (**b**) Expression analysis of *SmYTH1*–*SmYTH6* in leaves treated with 100 μM of SA for 0 h, 12 h, and 24 h. (**c**) Expression analysis of *SmYTH1*–*SmYTH6* in hairy roots with 100 μM of SA for 0 h, 3 h, and 6 h. *SmUBQ* was used as the normalization control. The values represent the mean ± SD of three biological and three technical replicates. The asterisks represent significant differences between the test group and the control group (0 h) (* *p* < 0.05, ** *p* < 0.01, and *** *p* < 0.001, Student’s *t* test), and ‘ns’ indicates no significant difference.

Under SA treatment, the expression of *SmYTH3* in roots was significantly downregulated at the time points of 12 and 24 h. *SmYTH5* and *SmYTH6* were downregulated at 12 h but upregulated at 24 h. *SmYTH2* was upregulated significantly at 24 h. The expression of *SmYTH1* and *SmYTH4* remained unaffected (Figure 12a). In leaves, the expression of *SmYTH3*, *SmYTH5*, and *SmYTH6* was significantly reduced at both time points. *SmYTH1* showed a significant reduction at 12 h but returned to the baseline level at 24 h. *SmYTH2* and *SmYTH4* exhibited no significant changes (Figure 12b). In hairy roots, SA treatment reduced the expression of *SmYTH2* and *SmYTH4*, whereas *SmYTH1*, *SmYTH5*, and *SmYTH6* showed downregulation at 3 h but returned to baseline levels at the time point of 6 h. *SmYTH3* was upregulated after SA treatment for 6 h (Figure 12c). These results indicate that some *SmYTH* genes also responded to SA treatment and potentially participated in SA-induced physiological and biochemical processes.

### 2.11. Increases of RA and Sal B Contents in S. miltiorrhiza Plantlets Treated with MeJA and SA

**Figure 13:**
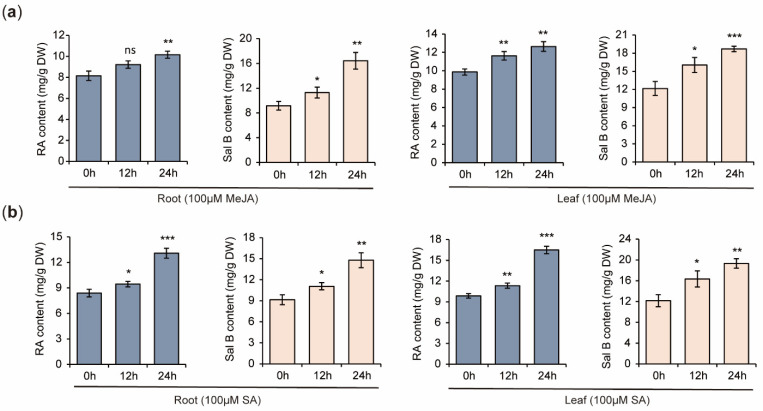
RA and Sal B contents in *S. miltiorrhiza* plantlets treated with MeJA and SA, respectively. (**a**) The contents of RA and Sal B in roots and leaves treated with 100 μM of MeJA for 0 h, 12 h, and 24 h. (**b**) The contents of RA and Sal B in roots and leaves treated with 100 μM of SA for 0 h, 12 h, and 24 h. The values are the mean ± SD of three biological and three technical replicates. The asterisks represent significant differences between the test group and the control group (0 h) (* *p* < 0.05, ** *p* < 0.01, and *** *p* < 0.001, Student’s *t* test), and ‘ns’ indicates no significant difference.

In order to elucidate the relationship between *SmYTH* expression and bioactive compound biosynthesis, the contents of RA and Sal B in *S. miltiorrhiza* plantlets treated with MeJA and SA were determined using HPLC. The results showed that MeJA and SA treatments caused significant increases of RA and Sal B in both roots and leaves (Figure 13). This suggests a negative correlation between the expression of various *SmYTH* genes, such as *SmYTH3*, and phenolic acid biosynthesis.

## 3. Discussion

Increasing evidence shows functional versatility of YTH proteins across plant species. For instance, members of the *Arabidopsis* ECT family are involved in plant morphogenesis, ABA signaling, flowering, and responses to salt and drought stresses [15,21,39,40,41,59]. Apple YTP2 protein may enhance plant resistance to powdery mildew [30]. Tomato SlYTH2 protein negatively regulates tomato fruit aroma [44]. Rice YTH07 may regulate flowering time [42]. Characterization of the *YTH* gene family in *S. miltiorrhiza* could provide foundational information for elucidating the biologic function of m^6^A and its reader proteins in this medicinal plant species.

Previous results showed that *A. thaliana*, tomato, alfalfa, and *Ginkgo biloba* harbored 13, 9, 53, and 10 *YTH* genes, respectively [25,60,61,62]. The deduced proteins spanned in all four clades, including YTHDF-1–YTHDF-3 and YTHDC [25,60,61,62]. However, no YTHDC clade members were identified in *S. miltiorrhiza*. The absence of YTHDC clade members could have resulted from genomic rearrangements, gene mutations, or gene deletions. The consequence of lacking YTHDC members is unknown. In addition, chromosome localization of *YTH* genes has been conducted for various plant species, such as *G. biloba* [62], *Cinnamomum camphora* [63], alfalfa [61], *Liriodendron chinense* [64], strawberry [65], cotton [66], and *Solanum lycopersicum* [67]. A cluster of multiple YTH genes has not been identified before. The cluster of *SmYTH8*–*SmYTH19* on chromosome 8 that was identified in the study could have resulted from gene duplication during species evolution. This duplication may enhance functional redundancy or diversification of SmYTHs. Furthermore, an F-box protein interaction domain was found for the first time at the N-terminus of SmYTH5–SmYTH19 proteins. F-box proteins are known to mediate ubiquitination and proteasomal degradation [68,69]. This indicates that these SmYTH proteins could interact with F-box proteins to regulate RNA stability or protein turnover, adding a novel layer of regulatory mechanisms to m^6^A-mediated post-transcriptional regulation. The unique distribution of *SmYTH* genes and the structural features of SmYTH proteins indicated that gene duplication and diversification play a critical role in the evolution of *SmYTHs* in *S. miltiorrhiza*. These unique features of SmYTHs indicated the significance of SmYTHs and functional difference of SmYTHs with the YTHs in other plant species.

Analysis of the crystal structures of the five animal YTH domain-containing proteins, including YTHDF1, YTHDF2, YTHDF3, YTHDC1, and YTHDC2, revealed that the YTH domain contained a well-characterized, conserved aromatic cage [34]. The cage was composed of either three conserved tryptophan residues (WWW) or two tryptophan residues and one tyrosine residue (WWY), and a positively charged concave structure rich in lysine and arginine. It facilitated the recognition and binding of m^6^A-modified RNA [31,54,70]. Among the 19 SmYTHs, SmYTH1–SmYTH4 proteins possessed the conserved “WWW cage” essential for m^6^A recognition. This feature was absent in SmYTH5–SmYTH19 proteins. In addition to the YTH domain, there was an N-terminal low-complexity region (LCR) in YTHDF proteins. This region, also called prion-like domains (PrLD), was the main factor initiating liquid–liquid phase separation (LLPS) in RNA-binding proteins [17,19,35]. Among the 19 SmYTHs, SmYTH1–SmYTH4 proteins were predicted to contain prion-like domains (PrLDs) at their N-terminals. This enabled phase separation—a mechanism critical for m^6^A-dependent regulation in both animals and plants (Figure 6). SmYTH1–SmYTH4 proteins that have the conserved YTH domain and PrLDs likely represent ancestral YTH proteins involved in fundamental m^6^A-dependent processes. Differently, SmYTH5–SmYTH19 proteins that have the additional F-box protein interaction domain could be evolved to fulfill specialized roles in leaf-specific processes. This evolutionary divergence highlights the adaptability of YTH proteins in different plant tissues and developmental stages.

Gene expression patterns are generally associated with gene functions. Analysis of the expression of *SmYTH* genes showed distinct spatiotemporal patterns. Among them, the expression level of *SmYTH3* in roots was significantly higher than other tissues. This indicated that this gene could play a dominant role among the 19 *SmYTH* genes. Consistently, SmYTH3 protein contained three PrLD regions that promote phase separation [15,40,42]. In addition, plant growth regulators played significant regulatory roles in the biosynthesis of bioactive compounds in *S. miltiorrhiza* [71]. The root of *S. miltiorrhiza*, as the primary medicinal part, contains medicinal ingredients, such as tanshinones and phenolic acids, which are induced by MeJA and SA [72,73]. This study showed that most *SmYTH1–SmYTH6* genes in roots and leaves had high sensitivity to MeJA and SA treatments, with the majority being downregulated (Figure 11 and Figure 12). On the contrary, the contents of phenolic acids were increased (Figure 13). The results indicated that SmYTH proteins could negatively regulate the biosynthesis of MeJA- and SA-induced phenolic compounds, highlighting the potential of *SmYTH* genes in the regulation of bioactive compound biosynthesis in *S. miltiorrhiza* through the mediation of plant growth regulator signaling pathways or direct involvement in metabolism of m^6^A transcripts encoding bioactive compound biosynthesis-related enzymes. In addition, the increases of RA and Sal B observed in this study were moderate, although the changes were statistically significant. Generally speaking, increasing the elicitor concentration and exposure time can enhance the impact of elicitors on metabolite accumulation, since the accumulation of metabolites is highly dependent on the elicitor concentration, exposure time, and plant growth stage. For the biotechnological application of this study, elicitor concentration and exposure time could be further adjusted.

Taken together, we provided a comprehensive characterization of the *SmYTH* gene family in *S. miltiorrhiza*. Their structural diversity, evolutionary relationships, and responses to PGR treatments were elucidated. This indicated the importance of *SmYTHs* in plant development and secondary metabolism. The results not only enhanced our understanding of m^6^A-mediated RNA metabolism in medicinal plants but also offered valuable targets for genetic engineering and breeding efforts aiming at the improvement of *S. miltiorrhiza* quality, yield, and environmental adaptability.

## 4. Materials and Methods

### 4.1. Plant Growth Conditions, Hairy Root Cultivation, and Hormone/PGR Treatments

In vitro sterile plantlets of *S. miltiorrhiza* line shh were cultivated on 1/2 MS media in a light cultivation room at the Institute of Medicinal Plant Development, Chinese Academy of Medical Sciences in Beijing, China. The plants were grown at the following conditions: a 16/8 h day/night cycle, day/night temperatures of 25 °C/22 °C, 80% humidity, and a light intensity of 250 μmol m^−2^ s^−1^. For gene expression analysis, roots, stems, and leaves were collected from one-month-old plantlets. For treatments, one-month-old plantlets were sprayed with 100 μM of methyl jasmonate (MeJA) or salicylic acid (SA) and then cultivated for 0 h, 12 h, and 24 h, respectively. Roots and leaves were collected at each time point. Mature root tissues were collected from plants grown in soil for three years. The epidermis, phloem, and xylem from three mature plants were isolated. All tissue samples were immediately frozen in liquid nitrogen after harvesting and stored at −80 °C until use.

For hairy root induction, sterile leaf discs were inoculated with the disarmed *Agrobacterium tumefaciens* strain ACCC10060 following the protocol previously established [56]. Hairy roots were routinely sub-cultured every 30 days on solid 1/2 MS medium. Healthy and vigorous growth hairy roots were selected to establish a liquid culture system in 6,7-V liquid medium. Hairy roots cultivated for 60 days were treated with 100 µM of MeJA or SA for 0 h, 3 h, and 6 h, and then harvested at each time point. All collected hairy roots were immediately frozen in liquid nitrogen and stored at −80 °C for RNA extraction.

### 4.2. Identification of the SmYTH Gene Family

To identify the *S. miltiorrhiza SmYTH* genes, *Arabidopsis* and *O. sativa* m^6^A reader protein sequences were downloaded from the UniProt Protein Database (https://www.uniprot.org/, accessed on 17 July 2024) and used as a query to search for homologous genes in the genome assembly of *S. miltiorrhiza* line shh (NCBI BioProject PRJNA903271) using tBLASTn v2.14.0 with a cutoff *e*-value of 1 × 10^−5^. Amino acid properties, molecular weights, and the theoretical isoelectric point (p*I*) were determined using the ProtParam tool (https://web.expasy.org/protparam/, accessed on 21 July 2024).

### 4.3. Analyses of Phylogenetic Tree, Gene Structure, Conserved Motif, Conserved Domain, and Cis-Acting Element

The sequences of YTH proteins in *S. miltiorrhiza*, *Arabidopsis*, *G. max*, and *O. sativa* were aligned using ClustalW with the default parameters (Table S1). The phylogenetic tree of YTH family members in the five species was constructed with MEGA11 software using the neighbor-joining (NJ) technique with a bootstrap value of 1000 replications [53]. Gene structure analysis was performed by comparison of the coding sequence with the genome assembly of *S. miltiorrhiza* line shh [56]. Conserved motifs of SmYTH proteins were analyzed using the MEME (https://meme-suite.org/meme/tools/meme, accessed on 13 August 2024) [74]. Conserved domains were analyzed using the Batch CD-Search Tool (https://www.ncbi.nlm.nih.gov/Structure/bwrpsb/bwrpsb.cgi, accessed on 13 August 2024) [75]. The 2000 bp upstream of the start codon of *SmYTH* genes was analyzed for *cis*-acting elements on the PlantCare website (http://bioinformatics.psb.ugent.be/webtools/plantcare/html/, accessed on 20 August 2024) [76]. The files generated from the analyses of the gene structure, conserved motif, conserved domain, and *cis*-acting element were further visualized using the corresponding plugins in TBtools v2.210 [77].

### 4.4. Chromosome Localization and Synteny Relationship Analysis

A file containing the length information for all chromosomes of *S. miltiorrhiza* line shh was downloaded [56]. The positional information of *SmYTH* genes on the chromosomes was extracted from the GFF3 file [56]. The two files were then submitted to TBtools for analyzing the chromosome locations of *SmYTH* genes. To examine the synteny relationship of the orthologous *YTH* genes obtained from *S. miltiorrhiza* and other plant species, the genome sequence file and annotation files of *Arabidopsis*, *G. max*, and *O. sativa* were downloaded from the Ensembl Plants database (https://plants.ensembl.org/index.html, accessed on 1 September 2024). The files were subsequently submitted to the ‘one step MCScanX-super fast’ tool in TBtools for syntenic analysis [77]. The results of chromosome localization and synteny relationship analysis (Table S2) were visualized using TBtools software [77].

### 4.5. Structure Construction and Liquid–Liquid Phase Separation (LLPS) Prediction

A PDB file of the model protein structure was downloaded from the RCSB PDB homepage (https://www.rcsb.org/, accessed on 22 September 2024), and then submitted with the protein sequence file to ESPrint3.0 (https://espript.ibcp.fr/ESPript/cgi-bin/ESPript.cgi, accessed on 22 September 2024) for secondary structure analysis [78]. The 3D (three-dimensional) structure of SmYTH proteins was predicted by homology modeling using the SWISS-MODEL server (https://swissmodel.expasy.org/, accessed on 25 September 2024) [79]. The prion-like domain (PrLD) and the disordered region (IDR) associated with LLPS of SmYTH proteins were predicted using the prion-like amino acid composition tool (PLAAC; http://plaac.wi.mit.edu/, accessed on 27 September 2024) [80].

### 4.6. Subcellular Localization of SmYTH3 Protein

The coding sequences of *SmYTH3* were amplified and inserted between the *Sal*I and *Eco*RI restriction sites of the pCambia1305 vector. *eGFP* was inserted at the C-terminus of the *SmYTH3* cDNA. Vectors carrying 35S::*SmYTH3-eGFP* were introduced into *Agrobacterium* cells GV3101. Healthy tobacco leaves were infiltrated with *A. tumefaciens* suspension carrying the target vector. Leaf samples (0.5 cm × 0.5 cm) were observed under a laser confocal microscope (LSM710, Zeiss, Jena, Germany).

### 4.7. Gene Expression Analysis by Quantitative Real-Time PCR (qRT-PCR)

Total RNA was extracted from tissue samples using the Quick RNA isolation kit according to the manufacturer’s instructions (Huayueyang, Beijing, China). The first-strand cDNA synthesis was performed using 2 μg of total RNA with the TRUEscript 1st Strand cDNA Synthesis kit (OneStep gDNA Removal; Aidlab, Beijing, China). Real-time qPCR was carried out using 2× Sybr Green qPCR Mix (Aidlab, Beijing, China) under the following amplification parameters: 95 °C for 2 min, followed by 40 cycles of 95 °C for 15 s and 60 °C for 30 s. The analysis was performed on a CFX96 Touch™ Real-Time PCR Detection System (Bio-Rad, Hercules, CA, USA). The primers used are listed in Table S3. Relative expression levels were determined using the *SmUBQ* gene as the normalization control. A total of three biological replicates were performed for each treatment. A plant or the hairy roots in a flask represented a biological repetition. The 2^−ΔΔCT^ method was employed to calculate the gene expression levels. ANOVA (analysis of variance) was calculated using GraphPad Prism 5.0.

### 4.8. HPLC Determination of Phenolic Acids

Phenolic acids were determined by high-performance liquid chromatography (HPLC). Elicitor-induced and non-induced plantlets were harvested and dried. The dried leaves and roots (100 mg) were ground into power, extracted with 3 mL of 50% methanol, sonicated for 1 h, and then kept at room temperature for 24 h. The extractions were filtered using 0.22 μm Millipore Express PES membrane filters. Isolation of phenolic acids and HPLC analysis were performed, as reported before [50]. Phenolic acids, including salvianolic acid B (Sal B) and rosmarinic acid (RA), were quantified through comparison with the authentic standard curves.

## 5. Conclusions

In this study, we systematically characterized the YTH domain-containing protein family, a class of m^6^A readers, in the medicinal plant *S. miltiorrhiza*. A total of 19 *SmYTH* genes were identified and mapped to five chromosomes. Phylogenetic tree, gene structure, conserved domain, synteny, and phase separation were analyzed by the bioinformatic approach, revealing that *SmYTH1*–*SmYTH4* exhibited more typical characteristic m^6^A reader protein features. *SmYTH* genes showed differential expression patterns. *SmYTH1*–*SmYTH4* were highly expressed in roots and leaves. *SmYTH8*–*SmYTH19* genes on chromosome 8 were exclusively expressed in leaves. In roots, *SmYTH3* was the most abundantly expressed *SmYTH*. The majority of *SmYTH1*–*SmYTH6* in roots, leaves, and hairy roots were responsive to MeJA and SA treatments. This indicates that some *SmYTHs* could be involved in MeJA- and SA-related signaling pathways. This work identified candidate genes for quality improvement of *S. miltiorrhiza* and provided useful information for further studies on the biological functions of m^6^A and YTH proteins in *S. miltiorrhiza*.

## Figures and Tables

**Table 1:** Characteristics of the *SmYTH* gene family in *S. miltiorrhiza*.

Gene Name	Chromosome	Start	End	ORF (bp)	AA ^a^	Mw ^b^	p*I* ^c^
*SmYTH1*	Chr1	48,158,370	48,164,782	1827	608	66,859.19	5.15
*SmYTH2*	Chr5	26,492,103	26,496,935	2103	700	76,823.39	5.92
*SmYTH3*	Chr7	11,251,622	11,255,803	2097	698	76,342.98	6.9
*SmYTH4*	Chr8	50,414,449	50,420,087	1692	563	61,918.98	7.96
*SmYTH5*	Chr3	64,499,982	64,503,334	1785	594	68,037.9	5.17
*SmYTH6*	Chr3	64,383,621	64,386,316	1869	622	71,225.09	4.69
*SmYTH7*	Chr8	5,040,013	5,042,279	1725	574	65,632.12	5.28
*SmYTH8*	Chr8	2,540,335	2,542,918	1785	594	68,402.62	5.15
*SmYTH9*	Chr8	2,556,826	2,558,542	1797	598	69,114.55	5.29
*SmYTH10*	Chr8	2,565,385	2,567,524	1782	593	68,542.75	5.41
*SmYTH11*	Chr8	2,587,693	2,589,834	1782	593	68,542.75	5.41
*SmYTH12*	Chr8	2,601,514	2,603,695	1800	599	69,634.15	5.39
*SmYTH13*	Chr8	2,612,165	2,614,350	1800	599	69,678.27	5.56
*SmYTH14*	Chr8	2,628,473	2,630,842	1803	600	69,720.34	5.33
*SmYTH15*	Chr8	2,634,422	2,636,603	1800	599	69,531.02	5.37
*SmYTH16*	Chr8	2,638,250	2,640,615	1812	603	69,615.04	5.26
*SmYTH17*	Chr8	2,656,711	2,658,896	1800	599	69,678.27	5.56
*SmYTH18*	Chr8	2,659,911	2,662,254	1788	595	68,603.27	6.17
*SmYTH19*	Chr8	2,668,109	2,670,480	1803	600	69,720.34	5.33

^a^ AA: number of amino acids. ^b^ Mw: molecular weight. ^c^ p*I*: isoelectric point.

## Data Availability

The data are available in the article and its Supplementary Materials.

## Supplementary Materials

The following supporting information can be downloaded at: https://www.mdpi.com/article/10.3390/ijms26104645/s1.
